# Validity and reliability study of the quality of recovery scale in Turkish

**DOI:** 10.55730/1300-0144.5680

**Published:** 2023-05-03

**Authors:** Ümmühan KILIÇ, Dilek KIYMAZ, Esra SARAÇOĞLU, Bahadır YAZICIOĞLU

**Affiliations:** 1 Division of R&D and Projects, Samsun Provincial Health Directorate, Samsun, Turkiye; 2 Division of Education and R&D, Samsun Education and Research Hospital, Samsun, Turkiye; 3 Division of Health Tourism and R&D, Samsun Education and Research Hospital, Samsun, Turkiye; 4 Division of Family Medicine Samsun Education and Research Hospital, Samsun, Turkiye

**Keywords:** Patient outcome assessment, postoperative recovery, quality of recovery scale

## Abstract

**Background/aim:**

The aim of this study was to adapt the “Quality of Recovery-15 Scale”, developed to measure the postoperative recovery quality of individuals, into Turkish by carrying out validity and reliability studies.

**Materials and methods:**

This methodological study was conducted with a total of 150 patients who underwent surgery under general anesthesia between November 2021 and January 2022 in a training and research hospital in the Black Sea region. Data was collected from the patients through the face-to-face interview method before the operation, on the 24th and the 48th hour postoperatively. First, the linguistic validity of the scale and then the validity and reliability analyses were carried out. Construct validity, confirmatory factor, and reliability analyses were then performed.

**Results:**

The Cronbach’s alpha coefficient of the scale was 0.851. The Kaiser–Meyer–Olkin test for goodness of fit of the one-dimensional 14-item scale was 0.853 and Bartlett’s test was significant. The goodness of fit values of the scale were found to be RMSEA = 0.149, CFI = 0.769, and GFI = 0.745, and they were considered acceptable levels. The eighth item was removed from the scale, which had originally consisted of 15 items, because the item correlation coefficient of this item was <0.200.

**Conclusion:**

The “Quality of Recovery Scale” was found to be a reliable and valid scale that can be used to measure the quality of recovery after surgery in Turkish society.

## 1. Introduction

Postoperative recovery is a complex process involving many variables such as stress, anxiety, pain, minor complications, surgery, and anesthesia [1–3]. Postoperative patient recovery is related to morbidity, mortality, changes in physiological parameters, and readmission rates. However, this data represents only one aspect of postoperative recovery [4,5]. Pain, nausea, and vomiting the patient may experience, the complications that might occur, and the emotional state of the patient are among the factors that might affect the quality of recovery in the postoperative period [5–7].

The patient’s self-evaluation should be taken into account along with physiological variables to determine an appropriate postoperative recovery plan [3,8]. While some measurement tools are used that consider the patient’s mental and physical health as a whole [8], others assess the patient by focusing more on his or her experience during the postoperative recovery period [3,9,10].

The “Quality of Recovery Scale (QoR)”, which was first developed by Myles et al. in 1999 and consisted of nine items, was used to determine the recovery of patients in the postoperative period [11]. This scale was later expanded to 40 items by Myles et al. in 2000, and the QoR-40 was created [12]. The same scale was revised again by Stark et al. in 2013 and converted into the QoR-15, which consists of 15 items, including areas such as postoperative pain, nausea, vomiting, and self-care [1]. The scale has been translated into many languages following validity and reliability studies [6,7,13–21].

During the literature review, we were unable to track down any study conducted on the Turkish validity and reliability of the QoR-15 version of the Quality of Recovery Scale. Therefore, the aim of this study was to conduct reliability and validity studies of the scale in Turkish.

## 2. Materials and methods

### 2.1. The design and the setting of the study

This methodological and descriptive study was conducted to evaluate the psychometric properties of the “Quality of Recovery Scale” in patients who had undergone surgery.

### 2.2. Population and sample of the study

**Figure 1:**
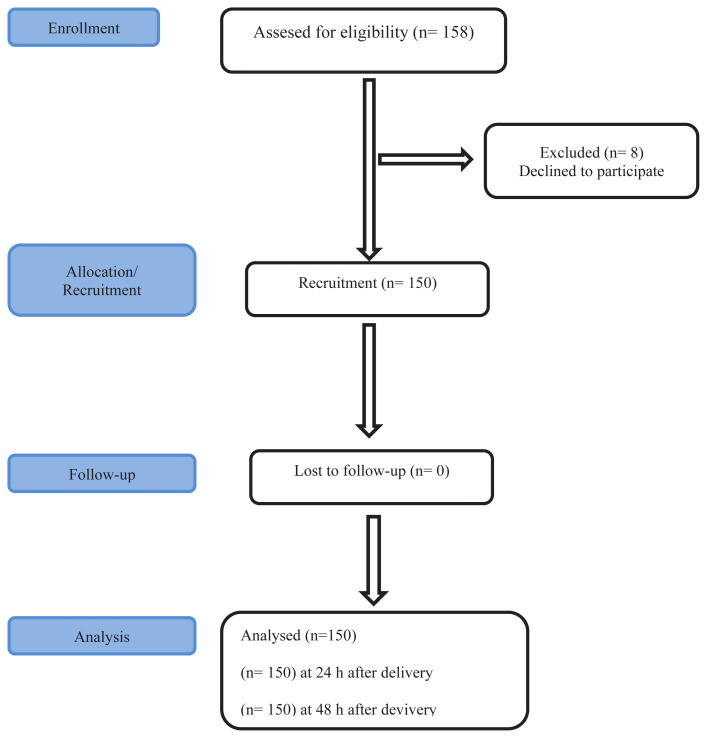
Flowchart of patient recruitment.

The study population consisted of patients who underwent surgery under general anesthesia at Samsun Training and Research Hospital between November 2021 and January 2022. While determining the sample size in scale adaptation studies, it is recommended to include a sample which is 10 times the number of items in the scale [22]. There are 15 items in the original scale for which validity and reliability studies would be performed. Therefore, based on 10 times the number of items in the scale, 150 patients were included in the study (Figure 1). The study included individuals who underwent surgery under general anesthesia, who were over 18 years of age, who were literate, whose health status did not prevent them from answering questions, and who voluntarily chose to participate.

### 2.3. Data collection tools

A questionnaire form was used to collect the study data. It consisted of the “Personal Information Form” and the “Quality of Recovery Scale.”

The Personal Information Form consisted of 15 questions prepared by the researchers regarding the participants’ demographic information and medical history.

The Quality of Recovery Scale (QoR-15) was developed by Stark et al. in 2013 to assess patients’ postoperative recovery status. It consists of 15 items that assess patients’ postoperative pain, nausea, vomiting, and self-care status. Each item is scored from 0 (never) to 10 (always). The 11th, 12th, 13th, 14th, and 15th items of the scale are reverse-scored. The total score of the unidimensional scale is calculated by summing the scores given for each item. The highest score that can be obtained from the scale is 150 and the lowest score is 0 (zero). A score close to 150 indicates that the quality of recovery is high [1]. The Cronbach’s alpha reliability coefficient of the scale, for which reliability and validity studies have been carried out in different countries, varies between 0.70 and 0.90 [1,4,7,19,20]. Furthermore, the scale was translated from English to Turkish using the translation/back-translation method, after which relevant experts were consulted and the language validity was carried out using the “Polit and Beck Content Validity Index” [23].

Study data was collected from the patients through face-to-face interviews at three different times: preoperatively, 24th hours postoperatively, and 48th hours postoperatively. A pilot study was conducted with ten patients to test the comprehensibility of the questionnaire, which was prepared by the researchers. There was no negative feedback from the participants of the pilot study. The data from the pilot study was not included in the study data.

### 2.4. Ethical aspect of study

The original version of the scale developed by Stark et al. in 2013 was obtained from the article entitled “Development and Psychometric Evaluation of a Postoperative Quality of Recovery Scale”. Permission to adapt the scale to Turkish was obtained from Paul S. Myles (one of the authors of Stark et al. (2023)) by e-mail.

Permission was obtained from the Clinical Research Ethics Committee of Samsun University (SUKAEK-2022/9/7) to carry out the study and it was registered on ClinicalTrials.gov (NCT05555368 09/22/2022). Verbal and written consent was obtained from the patients after they were informed about the study, and their participation was voluntary.

### 2.5. Evaluation of data

The study data was analyzed using IBM SPSS V23 and IBM AMOS. The Kolmogorov–Smirnov and Shapiro–Wilk tests were employed to test whether the data was normally distributed. The Mann–Whitney U test was used for the comparison of the paired-group data which was not normally distributed. The Kruskal–Wallis test was applied to compare three or more groups of data that were not normally distributed. Friedman’s test was performed to examine the temporal variation of the scale scores over time. In addition to these, multiple comparisons were performed utilizing Dunn’s test. Spearman’s rho correlation was used to examine the relationship between the data that did not have a normal distribution. Single-factor confirmatory factor analysis was applied in the confirmatory factor analysis of the scale, and the maximum likelihood method was implemented for the calculations. Internal consistency was assessed through item-total correlation and Cronbach’s alpha coefficient. In all analyses, p < 0.05 was accepted as the statistical significance value.

## 3. Results

The results of this study were presented as personal information, linguistic validity, content validity, construct validity, and reliability analyses of the scale.

In this study, the mean age of the patients was 50.70 ± 15.21 years. It was determined that 50% of the patients were female, 74% were married, 44.7% were primary school graduates, 67.3% were nonsmokers, 16.7% had heart disease before surgery, 46.7% had ASA II score, 45.3% had a history of previous surgery, and 22.7% had general surgery as their current type of surgery. The data is presented in Table 1.

No statistically significant difference was found between the median values of the total score on the Quality of Recovery Scale at 24 hours postoperatively according to sex, history of previous surgery, the status of ongoing medication uses for chronic disease, and type of current surgery. The relevant data can be observed in Table 2.

According to the Spearman correlation test, there was a negative statistically significant relationship between the total score on the Quality of Recovery Scale at 24 hours postoperatively and age (r: −0.221; p < 0.05).

### 3.1. Language and content validity of the scale

The translation/back-translation method was used to test the linguistic validity of the scale. The scale items were translated into Turkish by three experts. The scale items translated into Turkish were translated back into English by a native English-speaking expert who had no relationship with the subject and who lived in Türkiye. Then, to test the content validity of the Turkish translation of the scale, a content validity form was emailed to eight experts on the subject. The scores given by the experts for the items of the Quality of Recovery Scale were analyzed using Polit and Beck’s content validity index and it was found that there was a consensus among the experts. As a pilot study, the final version of the scale was applied to ten patients with similar characteristics to those to be included in the study. As each item on the scale was found to be understandable in the pilot study, no changes were made to the scale and validity/reliability analyses were continued.

### 3.2. Reliability

Cronbach’s alpha internal consistency coefficient, split-half, and item-total correlation analyses were carried out to test the reliability of the Recovery Quality of Recovery Scale.

Exploratory factor analysis was used to test the construct validity of the Quality of Recovery Scale. Exploratory factor analysis was employed to test the construct validity of the scale. The eighth item was removed from the scale because its correlation coefficient was less than 0.200.

Cronbach’s alpha coefficient was calculated to test the internal consistency of the scale. The Cronbach’s alpha value was 0.851 after removing the eighth item from the scale and it was found to have high reliability.

Furthermore, according to the results of the reliability analysis performed with the split-half method, the first half Cronbach’s alpha value was 0.682 at 24 hours postoperatively and 0.604 at 48 hours postoperatively. The second half Cronbach’s alpha value was 0.856 at 24 hours postoperatively and 0.938 at the 48 hours postoperatively. The Spearman–Brown coefficient was r = 0.600, and a high level of positive correlation was found between the sections. The relevant data is provided in Table 3.

In this study, the total score of the Quality of Recovery Scale was 94.57 ± 16.08 at 24 hours postoperatively and 109.87 ± 22.83 at 48 hours postoperatively. When the mean scores of the scale obtained at different times were analyzed, a statistically significant difference was found between them (114.667; p < 0.001) Table 4 displays this data.

### 3.3. Construct validity

In line with the answers given by the participants to the scale items, the Quality of Recovery Scale was made compatible with the data using confirmatory factor analysis. The measurement model established to confirm the single subdimension construct was determined by confirmatory factor analysis.

**Figure 2:**
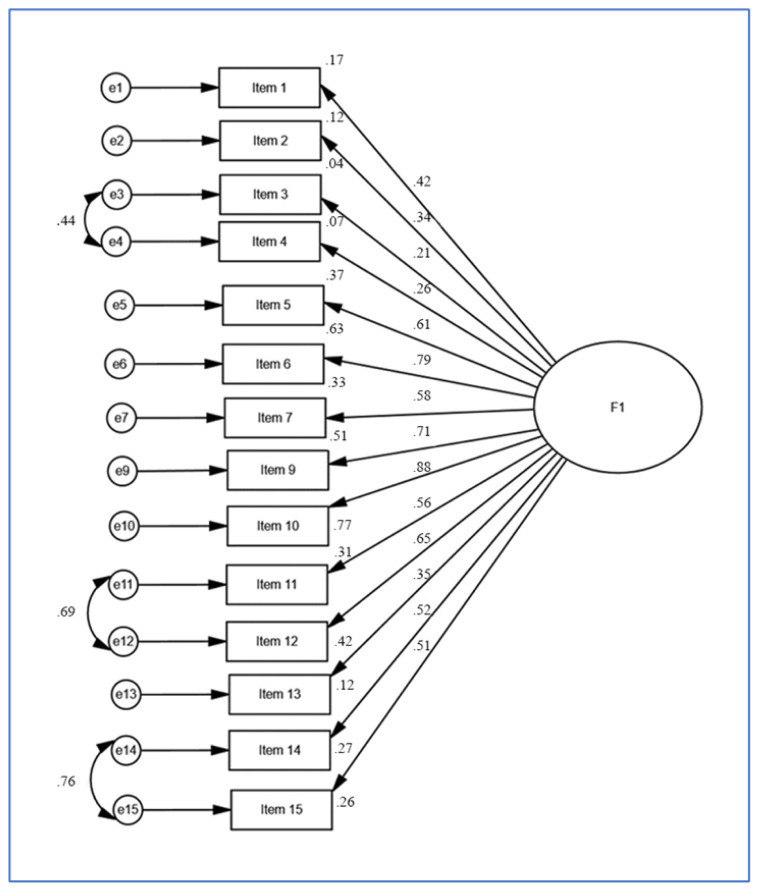
Standardized path coefficients of the quality of recovery scale.

The results of the analysis (model fit indices) of the QoR-14T scale are shown in Table 5. They indicate that the sample size was adequate for factor analysis. Furthermore, the high significance of the chi-square value for Bartlett’s test and the measure of sampling adequacy suggests that there was a significant relationship between the variables and that the data was suitable for factor analysis. In the measurement model of the scale, the factor loadings of all items were found to be at least 0.200. Accordingly, the tenth item can be said to be the strongest indicator of the scale (Figure 2).

## 4. Discussion

This scale is the first tool to assess the quality of postoperative recovery in the Turkish population. It is a measurement tool that relies not only on the assessment of healthcare professionals but also on the input from patients in assessing their recovery.

**Figure 3:**
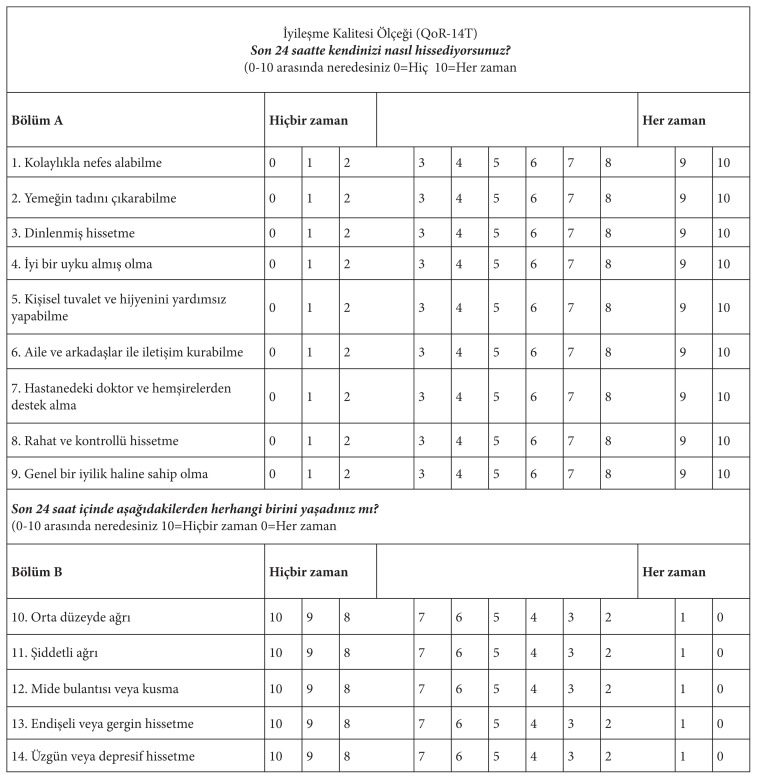
Final Turkish version of the OoR-14T scale.

One item was removed from the scale in the Turkish validity and reliability studies of the QoR-14T and, the new scale was found to have validity and reliability as the “Quality of Recovery Scale” (Figure 3). Furthermore, the reliability, responsiveness, acceptability, and ease of use of the QoR-14T scale were similar to the original validation, which was rated excellent [1].

When the reliability and internal consistency of the scale were evaluated, Cronbach’s alpha value of the Quality of Recovery Scale at 24 hours postoperatively was 0.851. In studies conducted in different countries using “QoR-15”, Cronbach’s alpha reliability coefficient values were found in the range of α = 0.076–0.81 [13,15,24]. Although it is sufficient to have a Cronbach’s alpha value of at least 0.50 in the scales, it has been noted that this value should be greater than 0.80 for high reliability [25]. Cronbach’s alpha value of this study was determined to be at a high-reliability level compared to the values of similar studies in the literature. QoR-14T can be considered a reliable and internally consistent assessment tool that can measure the quality of recovery after surgery.

In this study, the eighth item had a correlation value of less than 0.200. Therefore, it was removed from the scale. After this removal, the correlation coefficient values of the items were found to be between 0.291 and 0.721. According to the calculations, there was no negative correlation between the items. A positive and high item-total correlation indicates that the items have similar characteristics. In the QoR-14T scale, “*having a good night’s sleep*” was the item with the lowest correlation, while “*having a general well-being*” was the item with the highest correlation. While item 13 had the lowest correlation in the Japanese version, item 7 had the lowest correlation in the German version. These correlation coefficient values range between 0.27 and 0.82 [7,26]. Similar to studies in the literature, no negative correlation appeared to exist between items in the QoR-14T scale. According to the item-total correlation coefficients in this study, the reliability of the Turkish version of the scale was found to be high.

In the reliability analysis of the scale, the split-half method was used, and the first half of Cronbach’s alpha value (the first seven items) was calculated as 0.880 in the preoperative period, 0.682 at the 24th hour postoperatively, and 0.604 at the 48th hour postoperatively. The second half of Cronbach’s alpha value of the scale (last seven items) was 0.904 in the preoperative period, 0.856 at the 24th hour postoperatively and 0.938 at the 48th hour postoperatively. The Spearman–Brown coefficient was calculated as r = 0.600 for all items of the scale. These results indicate that the scale is reliable.

As a result of the Turkish validity and reliability analyses conducted in this study, the scale was determined to have a single subdimension as in the original [1] and some language adaptation studies [4,5,7,15,16]. There are also studies with two, four, and five subdimensions in the literature [17–19]. This might be because the scale was used in sample groups with different characteristics.

In this study, a negative statistically significant relationship was found between the total score of the Quality of Recovery Scale and age at the 24th hour postoperatively, whereas no relationship was found between age and the quality of recovery score in the studies conducted with the scale in UK and Portugal [1,14]. However, physiological changes occurring in the human body with age and frequent drug use, and an increase in chronic diseases affect the recovery process [27,28]. Some studies in the literature [29–31] show that postoperative recovery becomes more difficult with increasing age, an observation which supports our findings. This might be explained by the differences in the living standards of older adults in different societies, the variability of comorbidity with age, and the level of psychological and social care.

In this study, when the scores on the “Quality of Recovery Scale” were evaluated in the postoperative period, there was a difference between the Quality of Recovery Scale Scores between the 24th and 48th hours postoperatively, and it was found to be higher at the 48th hour postoperatively. Studies conducted in the UK, Portugal, and Germany with the scale measured the score only at 24 hours postoperatively [1,14,20]. This might have led to an underassessment of late complications or the level of recovery. Few studies in the literature have evaluated the quality of recovery scores both preoperatively and at the 24th and 48th hours postoperatively, as we did [32]. In our study, the QoR-14T score at 48 hours postoperatively was found to be very close to the preoperative score. This shows that evaluating patients with the QoR-14T scale at different time intervals after surgery is an accurate and effective method. The increase in the recovery score after 48 hours is thought to be due to the recovery beginning to take effect following the operation, the decrease in pain and nausea, and the patients starting to take care of themselves. Based on this finding, it can be said that the recovery of the patients tended to increase over time after the surgery.

This study has several limitations. First, we recognize that the sample size limited the ability to detect small differences. Second, we only included patients with eight types of surgery in the study. Third, as in previous studies, a single-center study was performed, possibly limiting the generalizability of the results.

In conclusion, our study showed that the QoR-14T is valid and has excellent reliability, responsiveness, and clinical feasibility as a measure of the quality of recovery in a Turkish surgical population. We believe that the QoR-14T is an appropriate scale for measuring health outcomes. Multicenter evaluation of the QoR-14T scale in different patient groups and larger samples is recommended.

## Figures and Tables

**Table 1:** Personal information of the participants.

		N	%
**Sex**	Female	75	50.0
	Male	75	50.0
Education level	Illiterate	9	6.0
	Literate	17	11.3
	Primary school	**67**	**44.7**
	High school	45	30.0
	University	12	8.0
Marital status	Married	**111**	**74.0**
	Unmarried	39	26.0
Smoking status	Yes	49	32.7
	No	**101**	**67.3**
Preoperative chronic diseases of the patient	Heart diseases	**25**	**16.7**
	Endocrine diseases	12	8.0
	Chest diseases	7	4.7
	Other	10	6.6
Status of continuous medication use due to chronic disease	Yes	45	30.0
	No	**105**	**70.0**
ASA* score	I	30	20.0
	II	**70**	**46.7**
	III	48	32.0
	IV	2	1.3
Status of previous surgery	Yes	68	45.3
	No	**82**	**54.7**
Type of the current surgery		**N**	**%**
	General Surgery	**34**	**22.7**
	Orthopedics	20	13.3
	Cardiovascular surgery	20	13.3
	Plastic surgery	19	12.7
	ENT	17	11.3
	Urology	17	11.3
	Neurosurgery	16	10.7
	Thoracic surgery	7	4.7
Age	**Mean ± SD**		
	50.70 ± 15.21		

* ASA (American Society of Anesthesiologists)

**Table 2:** Comparison of the personal information of the participants and the quality of recovery scale total score.

	Quality of Recovery Scale total scores at the postoperative 24th hour		Statistics	p
	X ± SD	Med. (min–max)		
**Sex**				
Female	94.57 ± 16.80	96.00 (55–131)	2399.50	0.120*
Male	109.87 ± 22.83	117.00 (51–203)		
**Status of previous surgery**				
Yes	92.75 ± 16.33	95.00 (58–126)	2343.50	0.139*
No	96.36 ± 16.84	99.00 (55–131)		
**Status of continuous medication use due to chronic disease**				
Yes	91.98 ± 14.91	91.00 (56–123)	1995.00	0.132*
No	95.69 ± 17.50	99.00 (55–131)		
**ASA score**				
I	99.73 ± 15.15	104.00 (65–120)		
II	96.14 ± 16.53	97.00 (55–131)	---	---
III	89.50 ± 17.29	90.00 (56–120)		
IV	84.00 ± 8.49	84.00 (78–90)		
**Type of the current surgery**				
Thoracic surgery	94.29 ± 9.25	90.00 (90–115)	11.801	0.107**
General surgery	93.18 ± 21.04	93.00 (58–123)		
Orthopedics	93.85 ± 16.23	99.00 (55–107)		
Neurosurgery	91.00 ± 17.48	89.50 (71–117)		
Cardiovascular Surgery	99.10 ± 11.37	99.50 (77–119)		
Plastic surgery	84.89 ± 15.76	77.00 (58–111)		
Ear nose throat	102.76 ± 16.62	105.00 (65–131)		
Urology	99.00 ± 11.36	103.00 (76–112)		

* Mann–Whitney U test;

** Kruskal–Wallis test, X ± SD (mean ± standard deviation)

**Table 3:** Exploratory factor analysis of quality of recovery scale items.

Scale items	Preop	Postop 24th hour	Postop 48th hour	Postoperative 24th hour						
				Before the item is removed				After the item is removed		
	Mean ± SD			r	D	α	α	r	α	α
1. Easy to breathe	8.82±1.52	7.78±1.59	8.52±1.59	0.436	0.669	0.826	0.834	0.426	0.845	0.851
2. Being able to enjoy the food	8.63±1.94	6.05±2.54	6.91±2.73	0.441	1.141	0.827		0.435	0.847	
3. Feeling rested	7.91±1.64	6.07±1.75	6.81±1.84	0.313	1.085	0.832		0.320	0.850	
4. Having a good night’s sleep	7.32±2.47	5.75±2.33	6.83±1.91	0.278	0.654	0.844		0.291	0.861	
5. Ability to perform toilet and personal hygiene needs without assistance	8.93±1.77	6.20±2.81	8.32±6.83	0.552	1.162	0.819		0.544	0.840	
6. Ability to communicate with family and friends	8.73±2.14	7.69±2.16	8.71±1.54	0.639	0.484	0.812		0.640	0.832	
7. Receiving support from doctors and nurses in the hospital	8.67±1.94	8.44±1.81	8.81±1.55	0.356	0.122	0.830		0.370	0.848	
**8. Ability to return to work or usual home activities**	5.39±3.42	2.83±2.01	5.05±1.98	**−0.007**	**0.912**	**0.851**				
9. Feeling relaxed and in control	7.78±2.52	5.49±1.64	7.15±1.91	0.670	1.077	0.815		0.661	0.834	
10. Having a general state of well-being	8.13±2.31	5.85±1.71	7.26±1.76	0.721	1.121	0.811		0.729	0.83	
11. Moderate pain	7.88±2.39	5.86±1.69	7.01±2.07	0.668	0.975	0.814		0.648	0.834	
12. Severe pain	8.45±2.51	7.23±2.12	8.43±2.08	0.670	0.525	0.811		0.682	0.83	
13. Nausea or vomiting	9.07±2.04	8.42±2.07	8.83±2.27	0.407	0.316	0.828		0.405	0.847	
14. Feeling anxious or nervous	6.68±2.27	6.53±2.05	7.87±2.44	0.504	0.069	0.822		0.529	0.839	
15. Feeling sad or depressed	7.18±2.26	7.21±2.07	8.41±2.15	0.516	−0.013	0.821		0.547	0.838	

Mean: Mean, SD: Standard Deviation, α: Cronbach alpha, r: correlation coefficient, D: Cohen’s effect size, Tukey summability test (F = 51.984; p = 0.301)

**Table 4:** Analysis of the temporal variation of the scores of the quality of recovery scale.

	Mean ± SD	Test Statistics	p
Preoperative	114.15 ± 22.34	114.697	**<0.001**
Postoperative 24th hour	94.57 ± 16.8		
Postoperative 48th hour	109.87 ± 22.83		

Avg: Mean, SD: Standard Deviation; Average: median; (min–max): minimum–maximum, Friedman

**Table 5:** Results of confirmatory factor analysis (model fit indices) of the scale.

Tests	Results
Kaiser–Meyer–Olkin measure of sampling adequacy	0.853
Bartlett’s test of sphericity chi-square	1955.050
Df (degrees of freedom)	136.00
Significance	0.000
χ^2^/df (The chi-square divided by the degrees of freedom)	4.2923
CFI (Comparative fix index)	0.769
GFI (Goodness of fit index)	0.745
RMSEA (The root mean square error of approximation)	0.149
